# The Effectiveness of 0.2% Chlorhexidine Gel on Early Wound Healing after Tooth Extraction: A Randomized Controlled Trial

**DOI:** 10.1055/s-0041-1739544

**Published:** 2022-01-11

**Authors:** Amaliya Amaliya, Rika Ramadhanti, Indra Hadikrishna, Tantry Maulina

**Affiliations:** 1 Department of Periodontology, Faculty of Dentistry, Universitas Padjadjaran, West Java, Indonesia; 2 Faculty of Dentistry, Universitas Padjadjaran, West Java, Indonesia; 3 Department of Oral Surgery, Faculty of Dentistry, Universitas Padjadjaran, West Java, Indonesia

**Keywords:** chlorhexidine gel, wound healing, tooth extraction

## Abstract

**Objective** This study aimed to evaluate the effect of 0.2% chlorhexidine (CHX) gel on wound healing after tooth extraction.

**Materials and Methods** A single blind, randomized controlled trial was performed recruiting 32 participants who underwent dental extractions. Patients were randomly allocated for CHX group or placebo group. The primary outcomes were wound closure measured with calipers and healings were assessed by Landry et al index after 7 days of topical application of allocated gels on extraction sites.

**Results** The wound closures were greater in CHX group compared with placebo group and healing scores were correlated with the use of CHX gel ( *p* -value < 0.05).

**Conclusion** In a population of healthy nonsmoker adults, application of 0.2% CHX gel twice a day for 7 days after tooth extraction has a beneficial effect on wound healing.

## Introduction

Tooth extraction is a very common procedure that dentists perform every single day. Postoperative complication generally do not occur; however, occasionally delayed wound healing may arise even in normal healthy patients. The most frequent postextraction complications documented were infection, prolonged bleeding, swelling, as well as dry socket. 1 In addition, patient may experience pain even after a simple uncomplicated tooth extraction. 2 3

Oral healing is slower and delayed compare with dermal repair. 4 Unlike skin surface, oral environment cannot be sterilized from oral bacteria or plaque formation, leading to persistent environmental challenge for the oral wound. Infection is one of the significant causes of delayed wound healing; therefore, early period of healing after tooth extraction must be facilitated and protected from infection or any condition inhibiting healing and repair. 5

After the procedure is performed, the site of tooth extraction may serve as a niche for bacterial plaque formation, due to the fact that patients cannot maintain good oral hygiene. 6 Patients may have hesitation to brush the wound site due to the pain or discomfort and may be instructed by dentist to avoid the brush to avoid hitting the extraction socket. To inhibit bacterial plaque accumulation, several interventions have been incorporated in postextraction procedures such as administration of antibiotics, mouthwashes, or topical medications, thereby preventing infection and chronicity of the wound. 7 8

Unfortunately, there is emerging situation of antimicrobial resistances caused by antimicrobial abuse, especially from systemic antibiotic use. To overcome this problem, topical antimicrobials in wound therapy are increasing in use. 9 10 11 In this method of therapy, topical antimicrobials are directly applied to the wound, resulting in a high concentration at the wound site, low systemic side effects, and a low incidence of antimicrobial resistance. One of topical antimicrobial agents recently available in the market is chlorhexidine (CHX) digluconate. Over a period of 40 years, CHX is widely used in dentistry and considered as an excellent antiplaque agent, due to its high substantivity and broad antimicrobial spectrum. Several forms of CHX have been prepared, that is, mouthwash, spray, chip, cement, varnish, dentifrice, and gel. 12 13 It has been found to have superior antimicrobial activity compared with other active agents. 14 Nevertheless, disadvantages remain prominent, it was found that CHX in the form of mouthwash showed the most common side effect of tooth restoration and tongue staining. 15 16 In addition, there is some evidence that regular and frequent application of CHX mouth rinses may temporarily impair the taste sensation, and promotes supragingival calculus formation and desquamative lesion of oral mucous. 17 Other disadvantage that may occur that is rinsing 24 hours after tooth extraction may dislodge blood clot that forms in the socket; therefore, a method of administration of CHX that may enhance and facilitate wound healing after tooth extraction is needed. 18

Topically applied CHX gel (Perio-Kin) has been proven to enhance wound healing in rats both at the clinical and histological levels without any adverse effect. 19 The use of the gel preparation had also been shown to reduce the incidence of dry sockets after third molar extraction; nevertheless, the method of treatment could only be performed by the surgeon. 20 The present study aimed to evaluate the effect of 0.2% CHX gel on early wound healing after tooth extraction of mandibular first molar, applied topically on the top of the wound site by the patients.

## Materials and Methods

The study design was a single blind, randomized controlled trial (RCT). The period of the trial was from August to December 2019, and the study protocol was registered in February 2021 at UMIN clinical trial registry with clinical trial registration number UMIN000043357. The participants consisted of 32 healthy nonsmokers who were about to undergo unilateral extraction of mandibular first molar at the Oral Minor Surgery Outpatients Ward of Dental Hospital, Universitas Padjadjaran, Bandung, West Java, Indonesia. Sample size calculation was performed by using the following formula: ( *r* − 1) ( *t* − 1) ≥ 15, where *t* is the number of groups and *r* is the number of samples.

Therefore, the number of subjects was 16 participants for each group.

Prior to the start of the study, ethical clearance was obtained from the Research Ethics Committee Faculty of Medicine Universitas Padjadjaran, Bandung, Indonesia (1497/UN6.KEP/EC/2018). Every procedure and ethical aspect of the current research has been conducted in full accordance with the World Medical Association's Declaration of Helsinki, and all participants gave their consent for their participation in the current study. Inclusion criteria of eligible participants were as follows: (1) patients who underwent tooth extraction of mandibular first molar, (2) aged 18 to 50 years with American Society of Anesthesiologists (ASA) physical status I, (3) did not have the medical history of being allergic to CHX and mefenamic acid, (4) was not consuming any other antibiotic, analgesic or anti-inflammation drug(s) at least 7 days prior to the procedure, (5) lack of infection at the tooth indicated for extraction 3 days prior to the extraction procedure, and (6) absence of any pathology at the area of the tooth indicated for extraction and neighboring teeth. Participants with medically compromised condition, being pregnant, or with tooth requires surgical extraction were excluded from the study.

Participants who fulfilled the inclusion criteria were then divided randomly into one of the following groups: the test group that received topical administration of 0.2% CHX digluconate gel (Perio-kin, Laboratorios Kin S.A., Barcelona, Spain) and 500 mg of mefenamic acid or the control group that received topical administration of placebo gel (Carboxymethyl Cellulose Sodium, glycerin, and aquadest) and 500 mg of mefenamic acid. Randomization was performed by taking a closed envelope containing the name of the group to which the participant was assigned to. The field researcher (T.M.) was responsible for this procedure and supervised each randomization procedure. After the participant was assigned to a group, the field researcher made the necessary record in confidential in the research database.

All participants underwent dental extraction under local anesthesia at the Oral Minor Surgery Outpatients Ward of Dental Hospital, Universitas Padjadjaran, Bandung West Java, Indonesia. The procedures adhered to the standard pre- and postoperative extraction protocols. Each removal of the mandibular first molar procedure was conducted by using 2 mL of lidocaine HCl with epinephrine as the local anesthetic and direct inferior alveolar nerve block as the injection technique. Mandibular first molars were extracted using simple extraction with close method, with No. 17 or No. 23 forceps. No. 17 forceps was seated as far apically as possible. Luxation of the molar was initiated with a buccal movement and then to the lingual. Subsequently, molar was delivered in the bucco-occlusal direction. No tooth sectionings were done during all procedures. No sutures have been applied to obtain wound healing by secondary intention. All the patients received postoperative advice on good oral hygiene and information on how to apply the topical gel. All extraction procedures were performed by a certified dental surgeon (I.H.) who was not informed about the group of which the participant was assigned to. All the patients were instructed to apply the assigned gel with cotton applicators onto the extraction wound site two times (every 12 hours) for 7 days, and take the mefenamic acid pill if necessary.

Assessments of wound diameter were performed immediately after extraction and 7 days after procedure, in bucco-lingual and mesio-lingual widths using a vernier caliper. Healing was assessed using the standardized index by Landry et al (1988). 21 Calculation was done by measuring the wound diameter differences between pre-extraction (Day 1) and postextraction (Day 7) assessments.

All measurements were performed under the same conditions by one calibrated examiner (R.R.) who was not informed about the group of which the participant was assigned to. Reproducibility measurements in 20 other postextracted patients, 8 hours apart, showed an intraclass correlation coefficient of 0.93 and 0.90 for wound diameter and healing index measurements, respectively.

All of the data were subjected for normality test (Lilliefors' test). For wound diameter analysis, pair *t* -test was used since it was normally distributed. Due to the fact that the data obtained from healing index were not normally distributed, Kendall's rank correlation (Kendall's tau) was employed to measure the association. Statistical significance was identified by setting the *p* -value ≤ 0.05. Statistical analysis was performed using SPSS version 23 from IBM, United States.

## Results

**Fig. 1:**
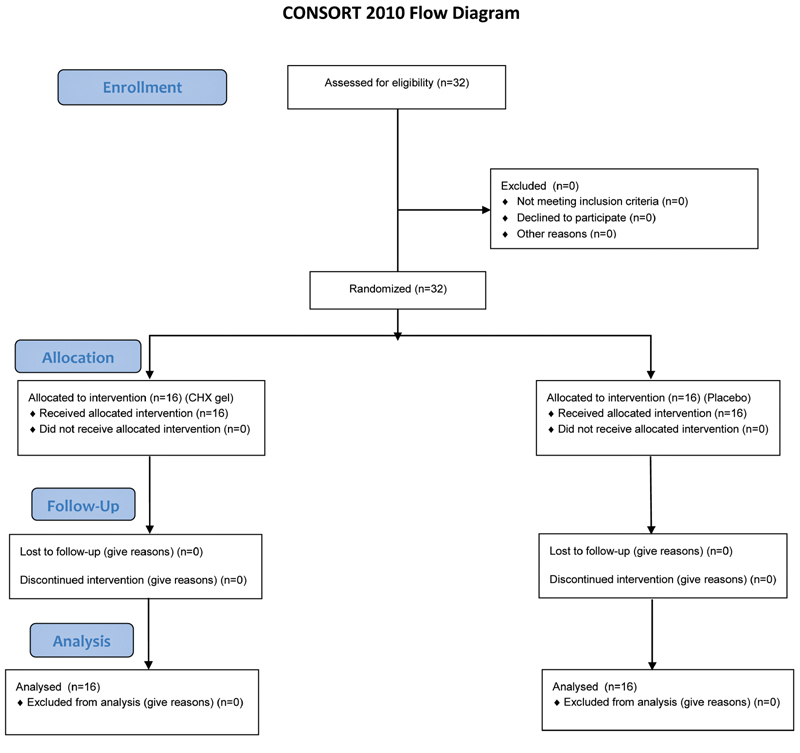
Consolidated Standards of Reporting Trials (CONSORT) flow of participants.

A total of 32 subjects (16 in test group and 16 in control group) completed the study. The phases of parallel RCT are shown in the Consolidated Standards of Reporting Trials (CONSORT) flow diagram ( Fig. 1 ). The mean age of the 32 subjects was 26.09 (±8.51) years without significant differences between the groups and ranged in all the two groups between 19 and 46 years. The study population consisted of 11 males and 21 females which was balanced distributed over the two study groups ( Table 1 ). No complication of extraction such as wound dehiscence, nerve disturbance, or prolonged pain was registered, nor allergic reaction was experienced by participants.

**Table 1:** Characteristics of participants

Variable	CHX	Placebo	*p* -Value
	*N* = 16	*N* = 16	
Age (y ± SD)	26.06 ± 9.45	26.13 ± 7.78	0.481
Sex (male/female)	7/9	4/12	0.457

Abbreviations: CHX, chlorhexidine; SD, standard deviation.

**Fig. 2:**
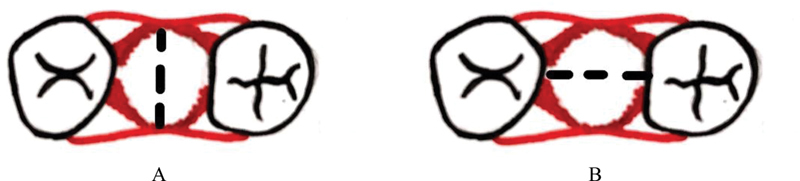
Wound diameter in buccolingual ( **A** ) and mesiolingual ( **B** ) direction.

In the present study, analysis of wound closure was performed by subtracting the wound diameter on Day 7 with wound diameter on Day 1 in buccolingual and mesiodistal directions ( Fig. 2 ). The mean values of wound diameter at different evaluation times are presented in Table 2 . The wound diameter in the test group decreased significantly in buccolingual, as well as mesiodistal direction compared with control group ( *p* -value < 0.05).

**Table 2:** Comparison of wound closure on Days 1 and 7 after tooth extraction

	X̄ (D1) ± SD (mm)	X̄ (D7) ± SD (mm)	∆ ± SD (mm)	*p* -Value
Buccolingual width				
CHX 0.2%	6.69 ± 1.42	2.41 ± 1.13	4.28 ± 0.65	0.0000000000637 a
Placebo	6.06 ± 1.81	3.91 ± 1.58	2.15 ± 0.60	
Mesiodistal width				
CHX 0.2%	9.25 ± 1.34	5.00 ± 1.22	4.25 ± 0.61	0.0000000000144 a
Placebo	8.40 ± 1,85	6.56 ± 1.80	1.84 ± 0.72	

Abbreviations: CHX, chlorhexidine; SD, standard deviation.

Notes: X̄ (D1) = mean wound diameter on Day 1. X̄ (D7) = mean wound diameter on Day 7. ∆ = wound diameter on D7 − D1.

a Significant difference.

Clinical features of wound healing were assessed on Day 7 by the use of Landry et al index (1988) ( Table 3 ). The frequency of healing scores experienced by subjects is presented in Table 4 .

**Table 3:** Wound healing index (Landry et al, 1988)

Healing index	Tissue color	Bleeding on palpation	Granulation tissue	Incision margin	Suppuration
1—very poor Two or more signs are present	≥ 50% of red gingiva	Yes	Yes	Not epithelized, with loss of epithelium beyond incision margin	Yes
2—poor	≥ 50% of red gingiva	Yes	Yes	Not epithelized, with exposed connective tissue	No
3—good	25–50% of red gingiva	No	No	No exposed connective tissue	No
4—very good	< 25% of red gingiva	No	No	No exposed connective tissue	No
5—excellent	All pink tissues	No	No	No exposed connective tissue	No

**Table 4:** Results of wound healing index (Landry et al)

	Healing index	*N* = 32	
		CHX	Placebo
1	Very poor	0	0
2	Poor	0	0
3	Good	5	5
4	Very good	8	10
5	Excellent	3	1
N		16	16

Abbreviation: CHX, chlorhexidine.

Correlation between healing score and the use of allocated gel was performed with Kendall's tau analysis and is presented in Table 5 . It can be seen that the use of 0.2% CHX gel had significant correlation with healing scores ( *p* -value < 0.05).

**Table 5:** Correlation of healing scores and allocated gel

	*p* -Value	W
CHX 0.2%		
Width	0.00000493 a	0.764
Healing index		
Placebo		
Width	0.3232	0.071
Healing index		

Abbreviation: CHX, chlorhexidine.

Note: W = Kendall's coefficient.

a Significant difference.

## Discussion

Several local and general factors affect oral wound healing, such as trauma, thermal damage, ischemia, wound size and location, edema, and infection. Healing within the oral cavity is a critical aspect since it occurs in warm oral fluid containing millions of microorganisms. Therefore, infection frequently occurs leading to poor wound healing. The aim of the present study was to evaluate the effect of topical application of CHX gel twice a day performed by the patients on top of the postextraction wound site. Healing was defined as wound closure and standardized healing index evaluating clinical appearance.

The result of the present RCT showed that 0.2% CHX gel significantly improved wound healing at the clinical level. In the test group that was instructed to apply with 0.2% CHX gel every 12 hours for 7 days after extraction, greater wound closure and better clinical healing were achieved as assessed by standardized healing index. This positive effect on clinical healing was related to the use of CHX gel as revealed by correlation analysis. In a meta-analysis study, Mínguez-Serra et al (2009) found that 0.2% CHX gel administered twice a day for 7 days would be the best option for preventing alveolar osteitis after extraction. 21 In the form of mouth rinse, it was shown by Halabi et al (2018) that 0.12% CHX mouthwash was able to prevent alveolar osteitis in a population having risk of developing alveolar osteitis (previous surgical site infection, traumatic extraction, and tobacco smoking) after tooth extraction, whereas Hita-Iglesias et al (2008) found that CHX in the form of gel may decrease the incidence of alveolar osteitis after mandibular third molar extraction compared with mouth rinse (7.5 vs. 25%). 20 22 A recent meta-analysis of 0.2% CHX gel application intra-alveolar showed that it was effective in preventing alveolar osteitis after third molar extraction. 23 In this present study, we evaluate CHX gel considering that the method of administration of this gel has the main advantage of providing a greater bioavailability in the application area, and therefore, the medication has a more prolonged release. Furthermore, administration of CHX gel was self-administered by the participants, and not performed by operators, so this way of administration could be considered as home care treatment.

In addition, the majority of the studies evaluating the protective effect of CHX toward alveolar osteitis were conducted in surgery procedures with sutures to close the wounds. However, this present study was performed in patients having tooth extraction without sutures at the end of the procedures. Thus, the participants in this present study had open wounds, and no dressing were applied instead of the test gel or placebo. The results of this present study are in accordance with Palaia et al (2019) who investigated the effect of mouthwashes containing the combination of CHX and sodium hyaluronate, CHX alone and placebo in second intention wound healing after oral biopsy with laser and without sutures. 24 CHX was proven to have accelerating effect on wound healing and can be recommended as good support or adjuvant therapy after surgical procedures. Furthermore, in a recent meta-analysis study, Armond et al (2017) suggested that the use of intra-alveolar CHX gel after surgical removal of mandibular third molars reduces pain, edema, and trismus after the extraction of third molars. 25

None of the participants of the CHX group in the present study experienced adverse events or hypersensitivity reactions after applying the gel. This could be the result of screening for CHX hypersensitivity before the commencement of the study that only individuals without allergic or hypersensitivity reactions to CHX could be included as participants. Nevertheless, several publications reported adverse events associated with prolonged use of CHX mouth rinse, ranged from mild to severe reactions including taste changes, tooth/tongue/staining, itching mouth, sore mouth, and increased calculus, while acute reactions had been reported as skin rash, nasal congestion, shortness of breath, swelling of face/lips/throat, nausea, swollen glands, diarrhea, abdominal pain, as well as anaphylactic reactions leading to death. 17 26 27 One of the study by McCoy et al (2008) reported that these adverse events occurred in older adults with uncontrolled diabetes, while in the present study, the participants included were younger than 50 years and systemically healthy with ASA status I. 17 Therefore, careful monitoring of adverse reactions in patients using CHX is warranted particularly among those with multiple medical conditions and a history of allergies or breathing problems, and the clinicians must carefully and completely advise patients who use CHX, in any form, of possible side effects.

In the present study, smoker individuals were excluded because several studies demonstrated that smoking is associated with delayed healing, wound infection, and dehiscence. 28 29 30 Nicotine in cigarettes has vasoconstriction effect, which may predispose to thrombotic microvascular occlusion and consequent tissue ischemia. According to Heng et al (2007), dry socket was found common among smokers after dental extractions as results of fibrinolytic activity and reduced alveolar blood supply. 31 It should be remembered that this study was performed in a group of nonsmokers. Therefore, it is currently unknown to what extent the present results can be extrapolated to smokers.

A relevant action of CHX in promoting wound healing may be due to its ability to reduce the bacterial load on the wound. Bacterial infection is proven to have clinical significance in impairing wound healing since it interferes with the normal wound healing process by stimulating the body's defense through activation of inflammatory cells and mediators, which in turn destructs the granulation and the surrounding normal tissues. 32 If inflammation persists, it will impair therapeutic intervention as well as tissue regeneration. 33 Back to 1970, Lindhe et al demonstrated that one daily topical application of 2.0% to the teeth and gingiva was able to remove bacterial plaque and resolved gingival inflammation in dogs. 34 Nevertheless, several studies on CHX have shown considerable contradiction on its effect on wound healing. Despite its strong bactericidal action, *in vitro* studies showed that CHX negatively affected fibroblast and keratinocyte proliferation in a dose-dependent manner, 35 36 37 while *in vivo* studies demonstrated the opposites. 38 39 40 These contrary results might be due to the different cellular and molecular interactions occurring *in vitro* and *in vivo* . *In vivo* , CHX in the form of positively charged bisbiguanide can bind to different negatively charged sites, including mucous membranes, salivary pellicle on teeth, and titanium surfaces, as well as several components of the biofilm on the tooth surfaces, for example, bacteria, extracellular polysaccharides, and glycoproteins. 41 42 Therefore, the remaining amount of CHX molecules available to bind to and harm host cells in the wound is significantly reduced. 43

Third molar surgery study design is usually the most validated study model to evaluate the effects of antimicrobial products on the postoperative period after tooth extraction, 20 21 22 23 44 but since the administration of CHX gel in those trial was more complicated than what was performed in this present study, we showed that CHX gel could also be administered by the patients themselves without having surgeon applying the gel into the postextraction socket. The ease and convenient way of this administration may be considered as home care for patients who underwent noncomplicated extractions.

A limitation of the present study is the relatively short period of early wound healing and that we only registered wound healing, while the severity of pain experienced by the participants was not recorded.

## Conclusion

In conclusion, under limitation of the present study, it is confirmed that CHX gel has the beneficial effect in enhancing wound healing after nonsurgery tooth extraction and may be suggested as adjuvant treatment and home care. It merits further studies to evaluate the effect of CHX with the evaluation of clinical and radiographic examination after tooth extraction.
